# Treatment Outcomes of Proliferative vs. Non-proliferative Adult Lupus Nephritis: A 10-Year Follow-Up

**DOI:** 10.7759/cureus.16955

**Published:** 2021-08-06

**Authors:** Mohamed Zahab, Mohammed A Fouda, Yasser Elhendy, Amir Elokely, Mona Abdul Rahim, Ayman F Refaie, Sami Alobaidi, Ahmed Akl

**Affiliations:** 1 Nephrology Department, Urology & Nephrology Center, Mansoura, EGY; 2 Internal Medicine Department, Zagazig University, Zagazig, EGY; 3 Pathology Department, Urology & Nephrology Center, Mansoura, EGY; 4 Department of Medicine, College of Medicine, University of Jeddah, Jeddah, SAU

**Keywords:** systemic lupus, lupus nephritis, outcome in lupus, cyclophosphamide, mycophenolate mofetil, rituximab

## Abstract

Introduction

Systemic lupus erythematosus (SLE) is a systemic disease with clinically heterogeneous outcomes. Lupus nephritis (LN) is a common complication of SLE. LN impacts clinical SLE outcomes both directly, in the form of target organ damage, and indirectly, through the adverse effects of immunosuppressive therapy.

Patients & methods

The study included 402 SLE cases with biopsy-proven lupus nephritis who were under follow-up for the past 13 years at Mansoura Urology and Nephrology Center, Egypt. We studied the differences in outcome among various LN classes and between 275 proliferative cases and 102 non-proliferative cases.

Results

Class IV was the main LN class in our series with renal survival of 60% at 10 years. The major induction regimen after the first biopsy was cyclophosphamide. Mycophenolate mofetil was the main induction and adjunctive regimen after the second biopsy. The mean follow-up period was 6.7 + 5.2 years. Higher serum creatinine, proteinuria, activity, and chronicity indices were noted in proliferative LN. Patients suffering from proliferative lesions received higher immunosuppression and demonstrated higher morbidity than those with non-proliferative lesions. Remission was higher among the non-proliferative compared to the proliferative group.

Conclusions

Serum creatinine, proteinuria, and LN class were the most relevant prognostic factors for renal survival among Egyptian LN patients.

## Introduction

Studying systemic lupus erythematosus (SLE) in humans is challenging, as the disease affects several organ systems generating clinically diverse outcomes. The characteristic indication of SLE is a systemic autoimmune response. This is ascribed to abnormal T-cell signaling pathways, reduced thresholds for initiating immune cell responses, and malfunctioning tolerance mechanisms [1]. As regards gender, SLE is often called a "woman’s disease" secondary to the striking differences in sex-related prevalence [2]. Lupus nephritis (LN) is not an uncommon consequence of SLE and occurs in 45%-86% of SLE patients in Japan and 31%-65% of SLE patients in the United States and European countries [3].

The clinical outcome is influenced by LN both directly and indirectly. The former results in organ damage, whilst the latter arises immunosuppressive therapy having deleterious effects [4]. Based upon their elevated creatinine levels and activity renal index values, renal impairment is more severe in male patients with LN than in female patients with SLE [5].

In LN, the major immunological features consist of a loss of self-tolerance to autoantigens [6]. In addition, the anti-double-stranded DNA antibodies directed against nucleosomes contribute to the glomerular injury [7]. The International Society of Nephrology/Renal Pathology Society (ISN/RPS) histopathological classification of LN continues to guide therapy, and all classes of LN have recently undergone a shift in management [8-9]. After the kidney becomes involved in SLE, the prognoses for lupus patients decline. This is due to chronic kidney injury [10].

## Materials and methods

Patients and methods

The patients' cohort included 402 cases with biopsy-proven lupus nephritis. In this retrospective evaluation, we selected patients fulfilling American Rheumatology Association (ARA) criteria for the diagnosis of SLE [11] and were under follow-up at Mansoura Urology and Nephrology Center, Mansoura University, for the past 13 years. The LN diagnosis was based on evidence of LN, including (1) Significant proteinuria (proteinuria defined as >0.5 g/day) and/or (2) Abnormal high serum creatinine associated with urine cellular or granular casts. Renal biopsies were performed when clinical and laboratory signs of renal involvement were observed. The renal biopsies analysis included light microscopy and immunofluorescence. Histological assessments were based on ISN/RPS (2003) [12]. In addition, we studied the differences in outcome between males (n. 40) and females (n. 363) in addition to the differences in outcome between proliferative (n. 275) and non-proliferative (n. 102).

Complete response was considered once serum creatinine return to baseline values and a decline in the urine protein/creatinine ratio (uPCR) to ˂500 mg/g (˂50 mg/mmol) [13]. Partial response is defined as stabilization (±25%) or improvement of creatinine, but not a return to normal, in addition to a ≥ 50% decrease in uPCR. If nephrotic-range proteinuria (uPCR ≥3000 mg/g [≥300 mg/mmol]) was observed, improvement required a ≥50% reduction in uPCR and a uPCR ˂3000 mg/g (˂300 mg/mmol) [13]. A sustained 25% increase in creatinine is widely used to define treatment failure but has not been validated [13].

The treatment of LN consisted of different combinations of oral steroids, azathioprine, and intravenous cyclophosphamide (The Euro-Lupus regimen). Furthermore, some patients received mycophenolate mofetil (MMF), plasmapheresis, intravenous methyl-prednisolone, cyclosporine A, and rituximab, according to clinical indications. The majority of patients in the non-proliferative group received cyclosporin.

Statistical analysis

The data extracted from the study were analyzed using SPSS for Windows (release 16 SPSS Inc., Chicago, III). Qualitative data were formulated in cross-tabulation, and quantitative data were presented in terms of arithmetic mean and standard deviation. Bivariate techniques were used for the initial evaluation of contrast. Thus, the chi-square and Fisher's exact test were used to compare qualitative variable frequencies, and the unpaired T-test was used to compare the means of two quantitative variables. Multivariate analysis was done using a Cox regression analysis. A p-value <0.05 was considered significant.

## Results

The mean age of SLE onset was 24.50±9.9 years while the mean age of LN onset was 26.57±8.8 years. The time between SLE and LN was 24.47±68.6 months. Male patients constituted 10.2% of the total cases. Most of our patients were virology negative (93.5%) regarding hepatitis C antibody, hepatitis B surface antigen, and human immunodeficiency virus antibodies. The family history of SLE was positive in 9.5% of our cohort. Mean basal serum creatinine was 1.37±1.5 mg/dl, and proteinuria (mean+SD) was 4±2.7 gm/day. About 4% of our patients did not proceed for renal biopsy. The most common cause of avoidance of renal biopsy was antiphospholipid antibody syndrome (APLS) on warfarin (1%) and proteinuria <0.5 gm (Table 1).

**Table 1:** Demographic characteristics of lupus nephritis patients

	Lupus nephritis cases (No.=402)
Age (years): (M±SD)	33.24±9.8
Sex: No. (%)	
Male	41 (10.2%)
Female	361 (89.8%)
Body mass index (M±SD)	32.92±21.8
Locality: No. (%)	
Dakahlia	231 (57.5%)
Damietta	61 (15.2%)
Kafe-Elskeikh	42 (10.4%)
Gharbia	33 (8.2%)
Others	35 (8.7%)
Age at onset of SLE (years): (M±SD)	24.50±9.9
Age at onset of LN (years): (M±SD)	26.57±8.8
Time between onset of SLE and LN (month): (M±SD)	24.47±68.6
Virology: No (%)	
Negative	376 (93.5%)
Positive	22 (5.5%)
Missing:	4 (1%)
Family history of SLE: No (%)	
YES	38 (9.5%)
NO	363 (90.3%)
Missing	1 (0.2%)

**Figure 1:**
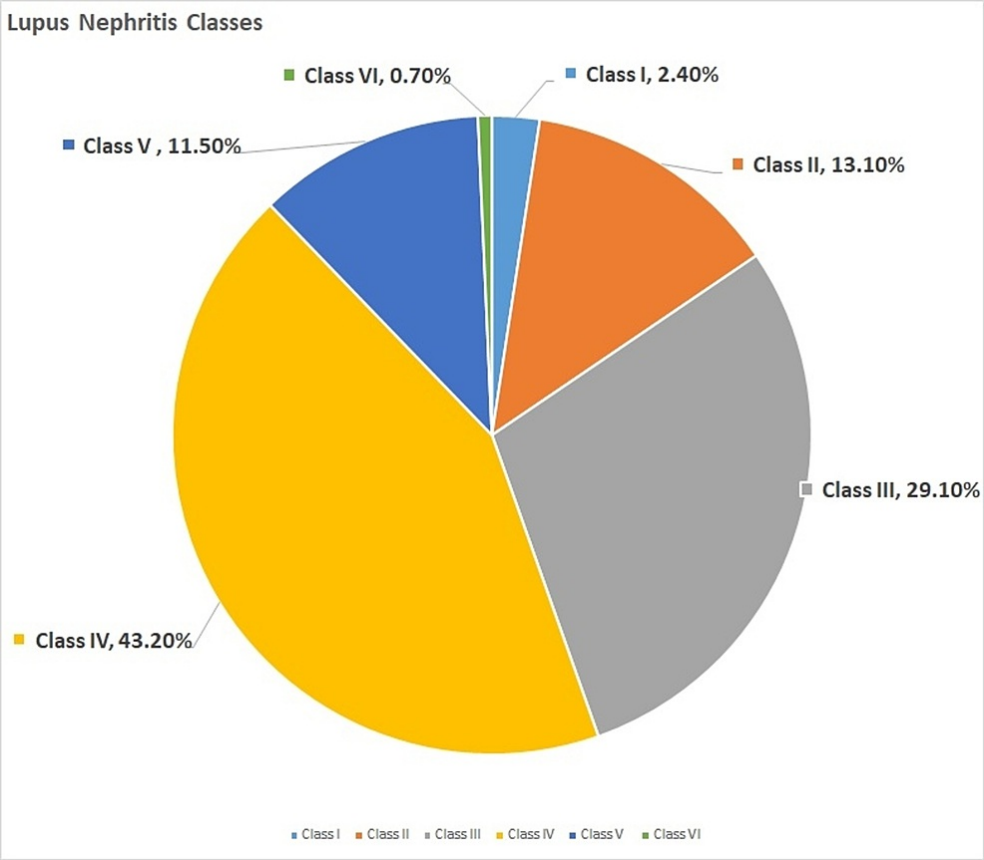
Prevalence of different LN classes among adult patients This chart demonstrates the prevalence of different histopathological classes among lupus nephritis (LN) patients.

**Figure 2:**
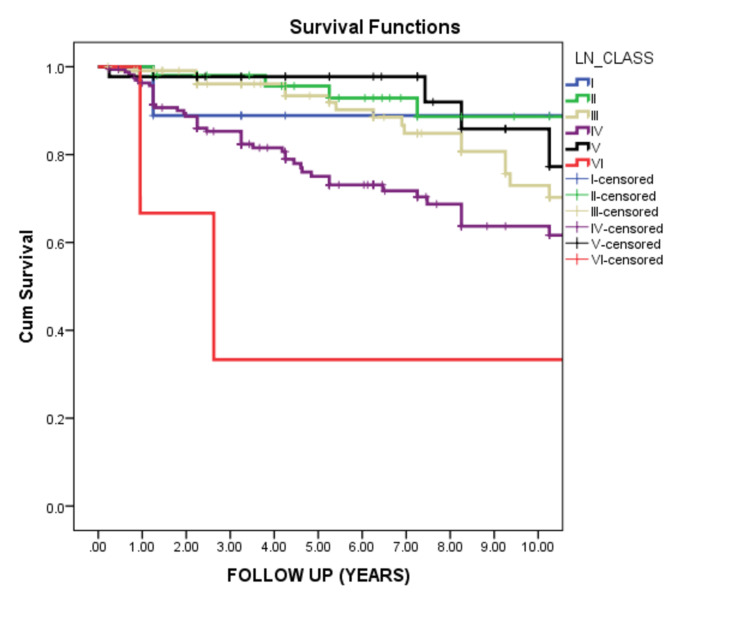
Survival of different LN classes among adult patients This curve demonstrated the significant difference in renal survival between different lupus nephritis (LN) classes. The best survival for classes 1,2,5 and the worst for classes 3,4,6. (P-value = 0.000)

Sixty-seven percent of our patients had proliferative LN. Class IV was the main LN class (43.2%) in our patient series. Class IV renal survival was 65% at 10 years, and class VI demonstrated the worst outcome (Figures 1-2).

About 22% of our patients received pulse steroids, and the rest received a daily dose of prednisolone 1 mg/kg of body weight (maximum dose 80 mg/day). Regarding induction therapy, 46% of patients received cyclophosphamide, and 19% received MMF. The primary induction regimen after the first biopsy was cyclophosphamide. Mycophenolate mofetil was the primary induction and adjunctive regimen after the second biopsy among LN patients.

Regarding maintenance therapy, 55.6% of our patients received steroid + azathioprine while 25.8% received steroid + MMF. Seventy-two percent of our cases received hydroxychloroquine. The mean follow-up period was about 6.7+5.2 years.

**Figure 3:**
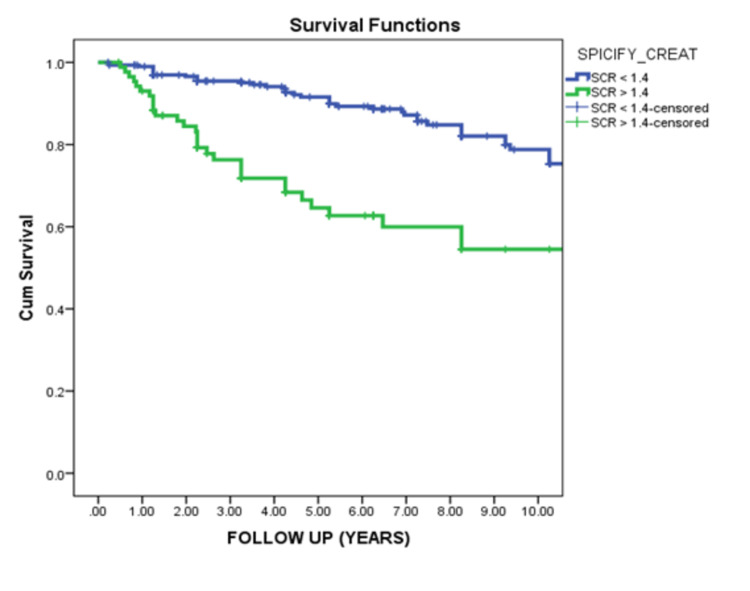
Renal survival in relation to the level of serum creatinine at first presentation This curve demonstrated that serum creatinine at the onset of the disease typically affects renal survival. Patients with normal serum creatinine (<1.4 mg/dl) showed significantly better renal survival than patients with high serum creatinine (>1.4 mg/dl) (p-value 0.000).

**Figure 4:**
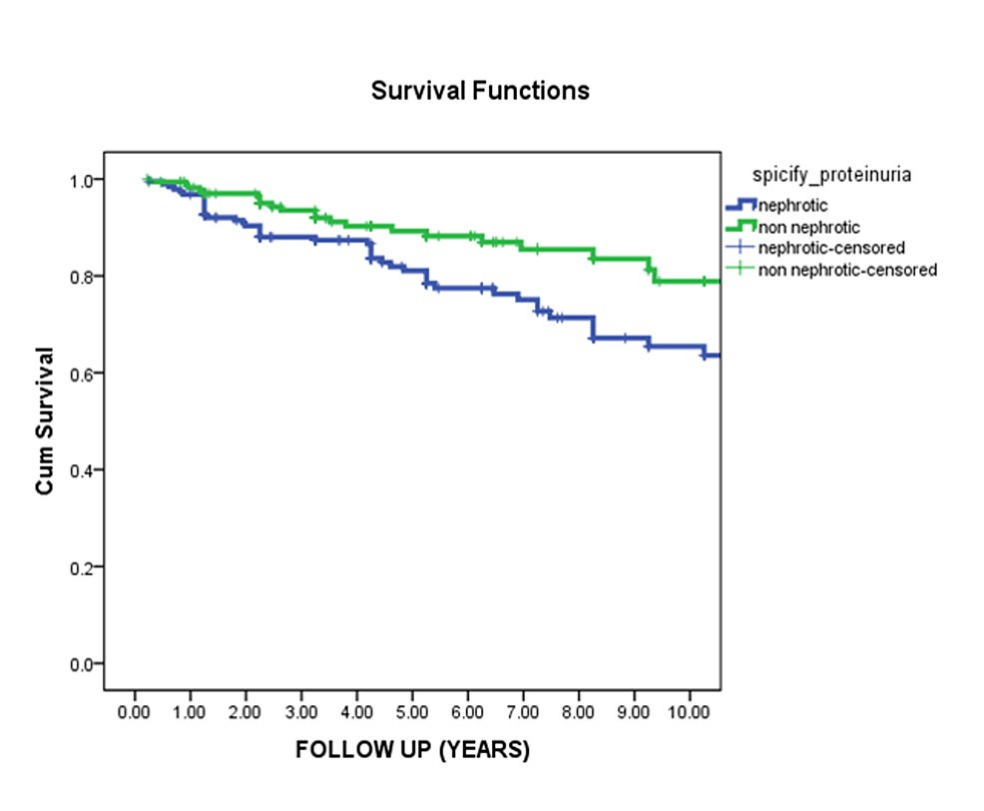
Renal survival in relation to the level of proteinuria at first presentation This curve demonstrated that the level of proteinuria at the onset of the disease typically affects renal survival. Patients with non-nephrotic range proteinuria showed significantly better renal survival than patients with nephrotic range proteinuria (p-value 0.006).

At first presentation, serum creatinine >1.4 mg/dl was associated with highly significant worse renal survival (P <0.001), and nephrotic range proteinuria was associated with the worst renal outcome (p=0.006) (Figures 3-4).

Hypertension and infection were the most common complications among our patients. APLS was seen in about 17% of our cases. Most of the in-remission cases were class I; also, most cases in relapse, CKD, and/or reaching hemodialysis were class IV cases. The highest percentage of partial remission was among class V and constituted 38% of our cases.

Table 2 shows the demographic characteristics of proliferative and non-proliferative lupus patients. Demographic characteristics were comparable in both groups. Proliferative LN was associated with statistically significant higher basal serum creatinine, basal proteinuria, and activity and chronicity indices.

**Table 2:** Demographic characteristics of proliferative and non-proliferative lupus nephritis patients

	Proliferative lupus nephritis (No.=276)	Non-proliferative lupus nephritis (No.=103)	P-value
Age (years): (M±SD)	33±9.8	33.38±9.2	0.7
Body mass index: (M±SD)	29.30±6.2	30.33±6.6	0.1
Locality: No. (%)			
Dakahlia	155 (56.1%)	60 (58.3%)	0.74
Damietta	42 (15.2%)	15 (14.6%)	
Kafe-Elskeikh	28 (10.1%)	13 (12.6%)	
Gharbia	21 (7.6%)	10 (9.7%)	
Others	30 (10.8%)	5 (4.8%)	
Age of onset of SLE (years): (M±SD)	24.81±10.54	24.12±7.85	0.26
Age of onset of LN (years): (M±SD)	26.65±8.78	26.31±8.22	0.21
Time between onset of SLE and L.N (month): (M±SD)	21.63±75.53	26.31±46.44	0.4
Virology: No. (%)			
Negative	261 (94.56%)	96 (93.20%)	0.39
Positive	15(5.43%)	7 (6.79%)	
Positive Family history of SLE: No.(%)	25 (9.1%)	11 (10.7%)	0.38
Positive Family history of renal disease: No.(%)	34 (12.3%)	10 (9.7%)	0.30
Complications: No. (%)			
Hypertension	221 (80.1%)	63 (61.2%)	0.001
Diabetes mellitus	25 (9.1%)	10 (9.7%)	0.4
Thrombotic events	36 (13%)	5 (4.9%)	0.01
Anti-phospholipid syndrome	50(18.1%)	10 (9.7%)	0.03
Neurological (stroke, sinus thrombosis, cerebritis)	31 (11.2%)	4 (3.9%)	0.01
Infection:	63 (22.8%)	14 (13.6%)	0.03
Pneumonia	41 (66.1%)	8 (57.1%)	0.1
Cellulitis	9 (14.2%)	2 (14.2%)	1.0
Intra-abdominal infection	8 (12.7%)	1 (0.07%)	0.08
Herpes zoster	4 (6.3%)	3 (21.4%)	0.07
Skin wart	1 (1.6%)	-	-
Malignancy	5 (1.8%)	2 (1.9%)	0.6

**Figure 5:**
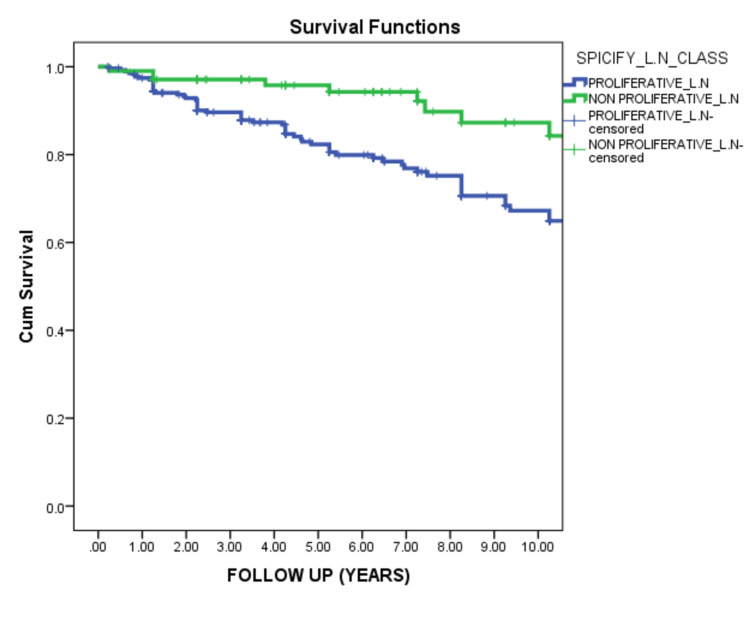
Renal survival of proliferative and non-proliferative lupus nephritis This curve demonstrated that the non-proliferative lupus nephritis group was significantly better in renal survival than the proliferative group (p-value = 0.001).

Proliferative LN was significantly associated with worst renal survival than non-proliferative LN (p=0.001) (Figure 5).

**Figure 6:**
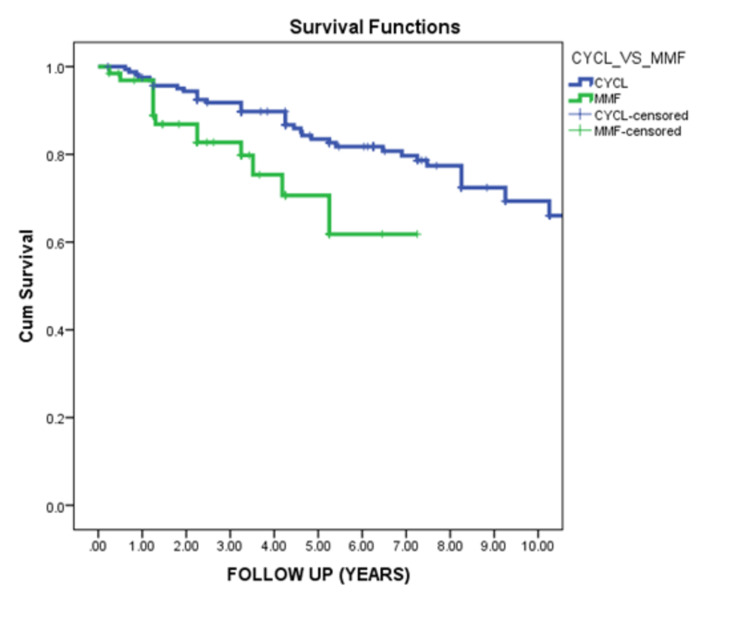
Renal survival in relation to the type of induction in proliferative lupus nephritis This curve demonstrated that proliferative lupus nephritis who received cyclophosphamide as induction therapy showed significantly better renal survival than patients who received MMF as induction therapy (p-value = 0.006). MMF: mycophenolate mofetil

Cyclophosphamide induction therapy compared to mycophenolate mofetil was associated with significantly better renal survival (Figure 6).

Forty-four percent (44%) of the proliferative and 33% of non-proliferative cases underwent second biopsy (p-value 0.3). Classes III and IV presented in proliferative LN lesions after the second biopsy to a much greater extent than in the non-proliferative lesions. Significant differences between both groups regarding the use of pulse steroids, induction, and maintenance therapies were noted. Hypertension [221 (80.1%) vs 63 (61.2%); p<0.001 ], APLS, infections, and thrombotic and neurological events were observed among the proliferative group (Table 2). CKD and end-stage renal disease (ESRD) were significantly higher among the proliferative group. Serum creatinine was higher at the last follow-up among the proliferative group over the non-proliferative group.

## Discussion

LN is an immune complex type of glomerulonephritis; patients with SLE often experience severe LN [14]. The inflammatory response caused by the accumulation of glomerular immune complexes initially damages the glomeruli, but with time, the damage extends to the renal interstitium. The prognoses for patients with LN deteriorate once the SLE affects the kidneys. Part of this deterioration is attributed to the onset of CKD or ESRD; there is also a heightened risk of CKD leading to cardiovascular disease.

In our study, renal survival among all LN patients was 95% after the first year, 86% after five years, 73% after 10 years, and 60% after 15 years. This was lower than reported by Al Arfaj et al., who reported high renal survival [15]. In a study from India, Dhir et al. reported that renal survival for Asian Indian patients was 79%, 70%, and 66% for five, 10, and 15 years, respectively [16]. Another study by Faurschou et al. found that five-, 10-, and 20-year renal survivals were 87%, 83%, and 73%, respectively [17]. Mok et al. found higher renal survival rates in patients with diffuse proliferative LN received cyclophosphamide [18]. Hypertension was the most common comorbidity found in lupus nephritis patients [19-20].

Proliferative LN was higher among male than female patients (79.5% versus 72.3%); however, the difference was not statistically significant (p=0.2). In addition, the male gender showed higher activity index in renal biopsies (p= 0.01). De Carvalho et al. reported that LN appears to cause greater renal damage as revealed by higher serum creatinine and activity renal index values in comparison with SLE in women [5]. Mahmoud et al. (2015) reported that the male gender is associated with poor outcomes [21]. In our study, male patients performed poorly regarding activity scores and kidney function but there were no differences in histology-based scores or renal replacement therapy requirements. Wang et al. reported similar data in Chinese males as compared to other nationalities [22].

In our data, proliferative LN showed significantly poorer renal outcomes than in non-proliferative LN patients (P=0.001). This goes hand in hand with Al Arfaj et al., who reported that proliferative LN is associated with poorer outcomes [14]. Also, Pinto et al. reported the aggressive behavior of proliferative nephritis [23]. Regarding induction therapy for proliferative LN patients, we found that induction with cyclophosphamide produced better renal survival than induction with MMF (P=0.006). In contrast, El-Shafey et al.'s data suggest MMF as an alternative treatment to induction by pulse cyclophosphamide [24]. However, Onishi et al. deferred the MMF superiority to pulse cyclophosphamide induction [25]. Kallenberg demonstrated that both the Euro-Lupus protocol and MMF with corticosteroids might be considered for remission induction in patients with proliferative LN [26]. Our data suggest that initial serum creatinine level at presentation carries significant predictive value to renal survival. High serum creatinine was found to be associated with poor renal outcomes [14,26].

## Conclusions

In conclusion, our study has the limitations of being a single-center study with a retrospective approach, hence prospective multicentre studies are needed to enforce evidence. Serum creatinine, proteinuria, and LN class are the most relevant prognostic factors for renal survival among LN patients. Management should be tailored according to proliferative or non-proliferative lupus nephritis to maintain efficacy and avoid side effects.
